# Coinfection with Human Herpesvirus 8 Is Associated with Persistent Inflammation and Immune Activation in Virologically Suppressed HIV-Infected Patients

**DOI:** 10.1371/journal.pone.0105442

**Published:** 2014-08-18

**Authors:** Mar Masiá, Catalina Robledano, Victoria Ortiz de la Tabla, Pedro Antequera, Blanca Lumbreras, Ildefonso Hernández, Félix Gutiérrez

**Affiliations:** 1 Infectious Diseases Unit, Hospital General Universitario de Elche, Universidad Miguel Hernández, Alicante, Spain; 2 Microbiology Service, Hospital Universitario de San Juan, Alicante, Spain; 3 Department of Public Health, Universidad Miguel Hernández, Alicante, Spain; University of Southern California Keck School of Medicine, United States of America

## Abstract

**Objectives:**

Infection with co-pathogens is one of the postulated factors contributing to persistent inflammation and non-AIDS events in virologically-suppressed HIV-infected patients. We aimed to investigate the relationship of human herpesvirus-8 (HHV-8), a vasculotropic virus implicated in the pathogenesis of Kaposi's sarcoma, with inflammation and subclinical atherosclerosis in HIV-infected patients.

**Methods:**

Prospective study including virologically suppressed HIV-infected patients. Several blood biomarkers (highly-sensitive C-reactive protein [hsCRP], tumour necrosis factor-α, interleukin-6, monocyte chemoattractant protein-1, vascular cell adhesion molecule-1, intercellular cell adhesion molecule-1, malondialdehyde, plasminogen activator inhibitor [PAI-1], D-dimer, sCD14, sCD163, CD4/CD38/HLA-DR, and CD8/CD38/HLA-DR), serological tests for HHV-8 and the majority of herpesviruses, carotid intima-media thickness, and endothelial function through flow-mediated dilatation of the brachial artery were measured.

**Results:**

A total of 136 patients were included, 34.6% of them infected with HHV-8. HHV-8-infected patients were more frequently co-infected with herpes simplex virus type 2 (HSV-2) (P<0.001), and less frequently with hepatitis C virus (HCV) (P = 0.045), and tended to be older (P = 0.086). HHV-8-infected patients had higher levels of hsCRP (median [interquartile range], 3.63 [1.32–7.54] vs 2.08 [0.89–4.11] mg/L, P = 0.009), CD4/CD38/HLA-DR (7.67% [4.10–11.86]% vs 3.86% [2.51–7.42]%, P = 0.035) and CD8/CD38/HLA-DR (8.02% [4.98–14.09]% vs 5.02% [3.66–6.96]%, P = 0.018). After adjustment for the traditional cardiovascular risk factors, HCV and HSV-2 infection, the associations remained significant: adjusted difference between HHV-8 positive and negative patients (95% confidence interval) for hsCRP, 74.19% (16.65–160.13)%; for CD4/CD38/HLA-DR, 89.65% (14.34–214.87)%; and for CD8/CD38/HLA-DR, 58.41% (12.30–123.22)%. Flow-mediated dilatation and total carotid intima-media thickness were not different according to HHV-8 serostatus.

**Conclusion:**

In virologically suppressed HIV-infected patients, coinfection with HHV-8 is associated with increased inflammation and immune activation. This might contribute to increase the risk of non-AIDS events, including accelerated atherosclerotic disease.

## Introduction

The spectrum of diseases causing morbidity and mortality in HIV-infected persons has shifted from AIDS-defining to non-AIDS related events, similar to those occurring in the general population [1]–[3]. This was related to the advent of combination antiretroviral therapy (ART), and its effects on immune dysfunction, lymphocyte activation and inflammation. However, excess morbidity and mortality do occur in HIV-infected compared to uninfected persons, and deaths from cardiovascular and liver diseases, non-AIDS defining cancer and suicide have been reported to be more frequent among patients with highly access to ART [4]. Different reasons have been implicated and, among them, a residual pro-inflammatory status despite effective ART [5], [6]. Several studies support increased immune activation and inflammation among virologically-suppressed HIV patients, and even in elite controllers, compared to the general population [7]–[10].

Infection with co-pathogens is one of the suggested mechanisms contributing to residual chronic inflammation after successful ART. Infections with the *Herpesviridiae* family are common among HIV-infected and uninfected persons. The relationship of many members of this family with cardiovascular disease has been assessed by measuring different markers of subclinical atherosclerosis and inflammation, and an association has been observed with cytomegalovirus, herpes simplex virus type 2 (HSV-2) and varicella-zoster virus in HIV-infected patients [11]–[13]. Human herpesvirus 8 (HHV-8) is a sexually transmitted pathogen highly prevalent among men who have sex with men [14], although additional transmission routes via saliva, infection during childhood, as well as by blood transfusion, have also been described [15], [16]. HHV-8 is a lymphotropic and vasculotropic herpesvirus linked with Kaposi's sarcoma, and possibly with pulmonary hypertension in HIV-infected patients [17]. Because of the ability of HHV-8-infected vascular endothelial cells to induce the expression of growth factors that cause angiogenesis, endothelial cell proliferation, enhanced vascular permeability, and cytokine production, it had been suggested that HHV-8 could be involved in atherogenesis, but data are very limited [18]. To explore its potential contributing role to the development of non-AIDS related events, and particularly of cardiovascular disease, we aimed to assess whether a relationship exists between HHV-8 and inflammation and subclinical atherosclerosis in virologically-suppressed HIV-infected patients. To that effect, we analyzed the relationship of HHV-8 with several biomarkers implicated in the pathogenesis of atherosclerosis, including markers of inflammation, endothelial activation, hypercoagulability, immune activation, and oxidative stress. We also evaluated the relationship of HHV-8 with carotid intima-media thickness (c-IMT), and endothelial function through flow-mediated dilatation measurement.

## Methods

We conducted a prospective study including consecutive stable HIV-infected patients on ART at increased risk for HHV-8 infection, cared for in an HIV outpatient clinic of a University Hospital in Elche, Spain. Accordingly, during a two-month period, adult (age ≥18 years) patients who had acquired the HIV through sexual transmission were invited to participate. Previous Kaposi's sarcoma was not an exclusion criterion. To avoid the confounding effect of HIV replication, only patients on ART with virological suppression, defined as a viral load <200 c/ml, were included in the study. Patients who agreed signed an informed consent before inclusion. The Hospital General Universitario de Elche Ethics Committee (CEIC) approved the protocol.

Clinical data were taken, and blood tests, c-IMT, and endothelial function were obtained after at least an 8 hour overnight fast and smoking abstinence. Self-assessment was used to check smoking abstinence; patients who recognized having smoked in the last 12 hours were given a new appointment for the flow-mediated dilatation test. A sample was processed by centrifugation. Plasma aliquots obtained were stored at −80°C.

### Biomarkers measurement

All frozen samples were subsequently defrosted, and plasma levels of vascular cell adhesion molecule-1 (VCAM-1), intercellular CAM-1 (ICAM-1), monocyte chemoattractant protein-1 (MCP-1), interleukin-6 (IL-6), tumour necrosis factor-alpha (TNF-α), plasminogen activator inhibitor (PAI-1), sCD14 and sCD163 were measured using commercially available ELISA kits (Quantikine, R&D Systems Europe Ltd, UK). Highly-sensitive C-reactive protein (hsCRP) was measured with a chemiluminescent immunometric assay (Immulite 2000, Siemens). D-dimer was measured with TECHNOZYM D-Dimer ELISA, TECHNOCLONE GMBH (Austria), and malondialdehyde (MDA) with HPLC analysis (CHROMSYSTEMS, Germany). CD4/CD38/HLA-DR and CD8/CD38/HLA-DR were measured with flow cytometry (BD FACSCalibur, BD Biosciences).

### Serologic tests for herpesviruses

IgG antibodies against the majority of the herpesviruses were measured by commercial ELISA test kits: FOCUS Diagnostics (Cypress, U.S.A) for HSV-1 and HSV-2; Trinity Biotech (Co Wicklow, Ireland) for Epstein-Barr virus; Vircell SL (Spain) for varicella-zoster virus; Panbio Ltd. Inverness Medical Innovations Australia Pty Ltd (Sinnamon Park, Australia) for HHV-6, and Siemens Healthcare Diagnostics (Spain) for cytomegalovirus. Results are provided in absorbance units (AU). The recommended cut-off point of >1.1 was considered positive, except for cytomegalovirus (>230 AU). For HHV-8 IgG antibodies detection, the Indirect Fluorescent Assay (ADVANCED BIOTECHNOLOGIES INC, USA) was used. This test provided qualitative, but not quantitative determination of IgG antibodies.

### Carotid intima-media and flow-mediated dilatation measurement

Endothelial function was evaluated by measuring flow-mediated dilatation of the brachial artery as detailed elsewhere [19]. cIMT measurement was performed as previously described [20]. Measures were taken from both common carotids and bulb portions. Total cIMT was calculated as the mean of all measurements. cIMT was analyzed as a continuous variable, and total cIMT was also categorized according to the median.

### Statistical analysis

Differences in demographic and clinical characteristics, in inflammation biomarkers, and in the surrogate atherosclerosis markers between patients with and without HHV-8 were assessed using the chi-squared or Fisher's exact test for categorical variables, and the Mann-Whitney test for continuous variables. The association of HHV-8 with hsCRP, the immune activation markers, and with the cIMT was assessed with linear regression models adjusted for the variables significantly associated with HHV-8 infection (P<0.05) and for the traditional cardiovascular risk factors: age, sex, hypertension, dyslipidemia, diabetes and smoking habit. Anti-platelet therapy was also included in the regression models. For regression analyses, all continuous variables were transformed on the natural log scale (log_e_) due to their highly skewed distribution.

## Results

157 patients on ART were screened; of them, 140 had an HIV viral load <200 c/ml, and in 136 serological results for HHV-8 were available. Baseline characteristics are shown in Table 1. Median (interquartile range) CD4 cell count was 652 (455–863) cell/mm^3^ and 634 (441–860) cell/mm^3^ in HHV-8 positive and negative patients respectively (P = 0.866). The most frequent antiretroviral regimens contained protease inhibitors (43.4% patients), and non-nucleoside reverse transcriptase inhibitors (NNRTIs) (39% patients); the majority (87.5%) of these patients were also receiving nucleoside reverse transcriptase inhibitors (NRTI), and 19 (17%) integrase inhibitors. Twenty one (15.4%) patients were receiving a raltegravir-based antiretroviral regimen (Table 1).

**Table 1:** Baseline characteristics of the patients by human herpesvirus 8 (HHV-8) infection serological status.

Variable	All patients (N = 136, 100%)	HHV-8 positive (N = 47, 34.6%)	HHV-8 negative (N = 89, 65.4%)	P
Age, years, mean (SD)	48.6 (11.6)	51.2 (13.4)	47.3 (10.4)	0.086
Sex, women	1 (0.7)	1 (1.1)	0	0.466
Race, Caucasian^a^	132 (97.1)	45 (94.6)	87 (97.8)	0.607
Duration of HIV infection	10.8 (5.4–15.7)	10.22 (5.3–15.1)	11.2 (5.4–16.0)	0.737
CD4 cell count, cell/mm^3^	650 (451–862)	652 (455–863)	634 (441–860)	0.866
PI-including regimen	59 (43.4)	18 (38.3)	41 (46.1)	0.409
NNRTI-including regimen	53 (39.0)	21 (44.7)	32 (36.0)	0.251
II-including regimen	21 (15.4)	7 (14.9)	14 (15.7)	0.79
Hypertension^b^	39 (28.7)	13 (27.7)	26 (29.2)	1
Dyslipidemia^b^	63 (47)	23 (50)	40 (45.5)	0.72
Diabetes^b^	10 (7.4)	1 (2.1)	9 (10.1)	0.16
Smoking	68 (50)	26 (55.3)	42 (47.2)	0.47
Lipid-lowering therapy	45 (33.1)	15 (31.9)	30 (33.7)	1
Anti-platelet therapy	19 (14.1)	7 (14.9)	12 (13.6)	1
Framingham risk score, %	6.5 (2.0–12.0)	7.5 (3–13.3)	6.0 (2–12)	0.248
Coinfection with other viruses				
Hepatitis C coinfection	12 (8.8)	1 (2.1)	11 (12.4)	0.045
Hepatitis B infection	9 (6.7)	4 (8.7)	5 (5.6)	0.497
Herpes simplex type 1	127 (93.4)	44 (93.3)	83 (93.6)	0.756
Herpes simplex type 2	76 (55.9)	38 (80.9)	38 (42.7)	<0.001
Varicella-zoster virus	132 (97.1)	46 (97.9)	86 (96.6)	0.683
Human herpesvirus 6	89 (65.4)	31 (66.0)	58 (65.2)	0.303
Cytomegalovirus	124 (91.2)	44 (93.6)	80 (89.9)	0.519
Epstein-Barr virus	135 (99.3)	46 (97.9)	89 (100.0)	0.167

Continuous variables are expressed in median (interquartile range), unless indicated. Categorical variables are expressed in no. (%).

PI, protease inhibitors; NNRTI, non-nucleoside reverse transcriptase inhibitors; II, integrase inhibitors.

a All non-Caucasian patients were people having origins in any of the original peoples of Central or South America.

b Hypertension, diabetes and dyslipidemia were defined by a previous diagnosis or by a current prescription of pharmacological therapy for any of such risk factors.

Serology tests showed that 34.6% patients were infected with HHV-8. Two patients had a history of previous Kaposi's sarcoma, and no patient had previous diagnosis of Castleman's disease. Prevalence of infection with other herpesviruses is shown in Table 1.

HHV-8 infected patients were more frequently co-infected with HSV-2 (P<0.001), and less frequently with hepatitis C virus (HCV) (P = 0.045), and tended to be older (P = 0.086) (Table 1).

### Relationship of Human herpesvirus-8 with biomarkers

Among blood biomarkers measured, HHV-8-infected patients had higher hsCRP levels (median [interquartile range], 3.63 [1.32–7.54] vs 2.08 [0.89–4.11] mg/L, P = 0.009), higher CD4/CD38/HLA-DR (7.67% [4.10–11.86]% vs 3.86 [2.51–7.42]%, P = 0.035), higher CD8/CD38/HLA-DR (8.02% [4.98–14.09] vs 5.02% [3.66–6.96]%, P = 0.018), and lower PAI-1 levels (6.60 [3.06–21.24] vs 15.23 [4.61–26.78] ng/mL, P = 0.034) than HHV-8-uninfected patients. Differences between most of the biomarkers remained after linear regression adjustment for the traditional cardiovascular risk factors, antiplatelet therapy, HCV and HSV-2 infection (adjusted difference between HHV-8 positive and HHV-8 negative [95% confidence interval] for hsCRP, 74.19% [16.65–160.13]; for CD4/CD38/HLA-DR, 89.65% [14.34–214.87]%; and for CD8/CD38/HLA-DR, 58.41% [12.30–123.22]% (Table 2). Only the differences in the levels of PAI-1 between HHV-8 groups lost the statistical significance after adjustment (−40.25% [−66.78–7.47]%).

**Table 2:** Relationship of human herpesvirus 8 (HHV-8) infection serological status with several plasma biomarkers, carotid intima-media thickness (cIMT), and flow-mediated dilatation of the brachial artery.

Variable	All patients (N = 136, 100%)	HHV-8 positive (N = 47, 34.6%)	HHV-8 negative (N = 89, 65.4%)	P	Adjusted difference between HHV-8 + and HHV-8 - (95% CI)^a^
Plasma biomarkers					
hsCRP, mg/L	2.51 (1.06–4.74)	3.63 (1.32–7.54)	2.08 (0.89–4.11)	0.009	74.19 (16.65–160.13)
IL-6, pg/mL	2.73 (0.88–7.24)	2.16 (0.82–6.34)	2.74 (0.90–7.21)	0.42	
TNF-α, pg/mL	19.7 (7.0–35.6)	24.05 (7.16–41.01)	17.38 (5.93–32.41)	0.38	
sICAM-1, ng/mL	64.8 (18.0–212.1)	71.0 (16.6–181.8)	60.6 (18.0–214.9)	0.58	
sVCAM-1, ng/mL	450.9 (143.2–739.0)	580.2 (152.4–848.4)	384.8 (138.4–687.5)	0.13	
MCP-1, pg/mL	687.8 (177.7–1374.4)	921.6 (159.8–1531)	604.8 (184.1–1228)	0.37	
CD4/CD38/HLA-DR, %	4.51 (2.75–10.43)	7.67 (4.10–11.86)	3.86 (2.51–7.42)	0.035	89.65 (14.34–214.87)
CD8/CD38/HLA-DR, %	5.43 (3.70–9.39)	8.02 (4.98–14.09)	5.02 (3.66–6.96)	0.018	58.41 (12.30–123.22)
PAI-1, ng/mL	13.25 (3.65–25.46)	6.60 (3.06–21.24)	15.23 (4.61–26.78)	0.034	−40.25% (−66.78–7.47)%
D-dimer, ng/mL	46.33 (25.23–68.01)	43.9 (23.6–61.5)	50.6 (26.3–77.5)	0.30	
MDA, µmol/L	0.13 (0.10–0.15)	0.12 (0.10–0.14)	0.13 (0.10–0.15)	0.78	
sCD14, pg/mL	5,5546 (3,196–13,834)	5,668 (3,883–14,928)	5,185 (3018–12,635)	0.18	
sCD163, ng/mL	942.5 (638.5–1,268)	983.9 (571.4–1,261)	912.8 (661.8–1,269)	0.87	
Total cIMT, mm	0.78 (0.65–0.98)	0.80 (0.67–0.97)	0.77 (0.64–0.99)	0.509	
Left bulb cIMT, mm	0.85 (0.70–1.14)	0.90 (0.75–1.15)	0.80 (0.69–1.10)	0.098	12.98% (−2.18–30.60)%
Right bulb cIMT, mm	0.90 (0.70–1.20)	0.95 (0.70–1.22)	0.90 (0.70–1.20)	0.60	
Carotid plaques (cIMT>1.5)	33 (23.57)	11 (23.40)	21 (23.60)	1.0	
Flow-mediated dilatation, %	4.04 (1.03–8.38)	5.12 (1.58–8.37)	4.0 (0.87–8.36)	0.54	

Continuous variables are expressed in median (interquartile range), unless indicated. Categorical variables are expressed in no. (%).

hsCRP, highly sensitive C-reactive protein; IL-6, interleukin-6; TNF-α, tumour necrosis factor-alpha; VCAM-1, vascular cell adhesion molecule-1; ICAM-1, intercellular CAM-1; MCP-1, monocyte chemoattractant protein-1; PAI-1, plasminogen activator inhibitor; MDA, malondialdehyde; cIMT, carotid intima-media thickness.

a Percent of difference between HHV-8 infected and uninfected patients after adjustment for HSV-2 and HCV infection, for the traditional cardiovascular risk factors (age, sex, hypertension, dyslipidemia, diabetes, and smoking habit), and for antiplatelet therapy. Percent of difference is calculated as 100 [e^mean difference from baseline^ -1] in which mean difference is calculated on the natural log scale. P<0.05 in all cases.

### Relationship of Human herpesvirus-8 with carotid intima-media thickness and flow-mediated dilatation

There were not differences in flow-mediated dilatation or total cIMT values between HHV-8-infected and uninfected patients. When cIMT at the different locations measured was analyzed, a trend to higher cIMT at the left carotid bulb was observed among HHV-8-infected patients (0.90 [0.75–1.15] vs 0.80 [0.69–1.10] mm, P = 0.098). This difference did neither reach statistical significance after linear regression adjustment (Table 2).

The relationship between inflammation biomarkers and subclinical atherosclerosis was explored. Subclinical atherosclerosis, defined as cIMT values above the median (0.78 mm), was associated with higher hsCRP (2.66 [IQR, 1.31–7.02] mg/L vs 2.07 [0.86–4.10] mg/L, P = 0.05). Subclinical atherosclerosis was also associated with higher sCD163 levels (1040 [806–1512] ng/mL vs 839 [578–1037] ng/mL, P = 0.004). No differences in the prevalence of HHV-8 infection were observed in patients with and without subclinical atherosclerosis (31% in those with cIMT<0.78 mm and 36.2% in those with higher values, P = 0.555).

## Discussion

This is the first study to explore the relationship of HHV-8 infection with inflammation and atherosclerosis in HIV-infected patients. We found that HHV-8 is associated with increased inflammation and immune activation, as reflected by higher levels of hsCRP, CD4/CD38/HLA-DR and CD8/CD38/HLA-DR. The surrogate markers of atherosclerosis assessed in the study, cIMT and endothelial dysfunction, were not different among HHV-8 infected and uninfected patients.

There was a strong association between higher hsCRP levels and HHV-8 infection. hsCRP is the best available cardiovascular risk predictor in head-to-head comparisons of various inflammatory markers, and the only biomarker considered to be an independent predictor of incident coronary heart/cardiovascular disease [21]. Additionally, hsCRP is an exquisitely sensitive and, currently, the more robust and best validated biomarker for low-grade systemic inflammation. In contrast to other systemic biomarkers, hsCRP is a highly stable molecule falling within a characteristic range for each individual, and it has also shown to remain highly stable when stored at −70°C [22]. Therefore, our findings support a pro-inflammatory state in virologically suppressed HIV-positive patients co-infected with HHV-8. Actually, inflammation induced by HHV-8-infected cells has been implicated in the pathogenesis of multicentric Castleman's disease and other viral-associated malignancies. Disease flares of multicentric Castleman's disease have been associated with elevated levels of a number of cytokines, including viral and human IL-6 or IL-10, and to a lesser degree of TNF-α [23]. Likewise, a systemic inflammatory syndrome in the absence of Castleman's disease has been described in HHV-8-infected patients in association with elevated viral IL-6 levels [24]. HHV-8 was also accompanied in our study by concomitant immune activation, as reflected with the elevation of CD4/CD38/HLA-DR and, to a lesser extent, of CD8/CD38/HLA-DR. Activated CD4^+^ and CD8^+^ T cells have been associated with subclinical carotid artery lesions in HIV-infected women [25].

Infection with other herpesviruses, specifically HSV-2, was also more frequent among the HHV-8-infected patients in our study, probably reflecting the same transmission route. Although HSV-2 has been associated with subclinical atherosclerosis in HIV-infected patients [13] and with elevated hsCRP levels [11], and a confounding effect could be therefore awaited, the relationship of HHV-8 with inflammation and immune activation remained after adjustment for HSV-2 and for the traditional cardiovascular risk factors. Likewise, the antiretroviral regimen composition could also have an impact in the biomarkers levels, since a pro-atherogenic profile with PI-based regimes, and anti-atherogenic with integrase inhibitors have been reported [26], [27]. However, the proportion of patients receiving the different ART classes in our study was comparable between HHV-8-infected and uninfected patients.

HHV-8 is a gammaherpesvirus with specific tropism for vascular endothelial cells. To date, the only available clinical data supporting a potential role for HHV-8 in atherosclerosis in HIV-infected patients come from a retrospective analysis of post-mortem reports describing a higher frequency of macroscopic atheromatous lesions in patients with Kaposi's sarcoma [18]. Despite the link of HHV-8 with inflammation, we could not demonstrate increased subclinical atherosclerosis through cIMT or endothelial function in HHV-8 coinfected patients. The low number of patients included in the study and, despite adjustment for cardiovascular risk factors and for other herpesviruses, the multifactorial and complex nature of atherosclerotic disease, probably precluded us from finding an independent relationship between HHV-8 infection and both atherosclerosis surrogate markers.

Limitations of the study are the cross-sectional nature of the associations studied, and the number of patients included, which might have been insufficient to show a statistical association between HHV-8 and subclinical atherosclerosis. Although indirect fluorescent assays are the most sensitive tests for HHV-8 detection, no single assay is completely sensitive and specific [28]. Therefore, misclassification of HHV-8 infected and uninfected patients might have occurred.

In summary, our study suggests that HHV-8 infection is associated with increased inflammation and immune activation in virologically suppressed HIV-infected patients. Our study might support a link between HHV-8 infection and inflammation as potential contributing pathogenic factor to the development of non-AIDS related events, including atherosclerotic disease, in HIV-infected patients.
